# Concurrent acute appendicitis and obstructive ureterolithiasis: a case report and review of literature

**DOI:** 10.1093/jscr/rjae576

**Published:** 2024-09-12

**Authors:** Dawood Alatefi, Abdulhakim M Hezam, Ahmed Alanzi

**Affiliations:** The University of Jordan Hospital, The University of Jordan, Amman 11942, Jordan; Orthopaedic Surgery, Salmaniya Medical Complex, Salmaniya 329, Bahrain; Anesthesia and Critical Care, King Hamad University Hospital, Muharraq 228, Bahrain

**Keywords:** appendicitis, case report, appendicolith, hydroureteronephrosis, obstructive hydronephrosis

## Abstract

The prediction of the coexistence of acute appendicitis and renal colic can be challenging, especially when the patient’s symptoms point toward one diagnosis. In this case report, we describe a patient who presented to the emergency department with severe lower abdominal pain that was thought to be solely due to acute appendicitis. Further evaluation, however, revealed the simultaneous coexistence of a right ureteral stone, causing severe hydroureteronephrosis. The patient underwent prompt surgical management, including laparoscopic appendectomy, ureteroscopy, and double-J stent insertion, and had an uneventful postoperative recovery.

## Introduction

Right lower abdominal pain is one of the most common surgical emergencies, with appendicitis being a frequent cause [1]. Renal colic due to a ureteral stone is also a common reason for this complaint. However, the simultaneous occurrence of these two acute pathologies is rare. To the best of our knowledge, only seven cases of acute appendicitis concurrent with a right ureteral stone and one case with a left ureteral stone have been reported in the medical literature [2–9]. Herein, we report a case of a male patient who presented with symptoms suggestive of appendicitis. Subsequent abdominal and pelvic computed tomography (CT) imaging revealed, however, the presence of right-sided ureterolithiasis in addition to acute appendicitis.

## Case presentation

A 30-year-old male patient presented to the emergency department with a severe right lower quadrant abdominal pain for the past 12 hours. He also reported experiencing nausea, a loss of appetite, and a single episode of vomiting. There had been no change in bowel or urinary habits since the onset of pain. His past medical history was unremarkable, and he had no prior surgeries or use of medications for chronic diseases. Upon physical examination, the patient was alert and oriented. The abdomen was soft and tender, with guarding and rebound tenderness noted in the right iliac fossa. The patient was afebrile and hemodynamically stable, but the blood pressure was elevated at 200/100 mm Hg. Laboratory workup was significant for leukocytosis (WBC 22.06 × 10^9^/L) with 89.60% neutrophils. The absolute neutrophil count (ANC) was elevated (20 × 10^9^/L). Creatinine was also noted to be at borderline (102 μmol/L). Urine dipstick and urine microscopy were unremarkable. Other laboratory values outside the reference ranges are shown in Table 1.

**Table 1:** Laboratory values.

Test	Result	Unit	Reference ranges
White blood cell (WBC) count	22.06	×10^9^/L	3.6–9.6
Neutrophils %	89.60	%	42.2–75.2
Neutrophils absolute count	20	×10^9^/L	2.3–8.1
Creatinine	102.00	μmol/L	65–104
Red blood cell count	5.96	×10^12^/L	3.9–5.2
Hemoglobin	16.80	g/dL	12.0–14.5
Hematocrit	49.00	%	33–45
Platelet size distribution	11.4	%	46–50
Eosinophils %	0.00	%	1.0–4.0
Globulin	33	g/L	15–30
Alanine aminotransferase	46	U/L	≤41

**Figure 1:**
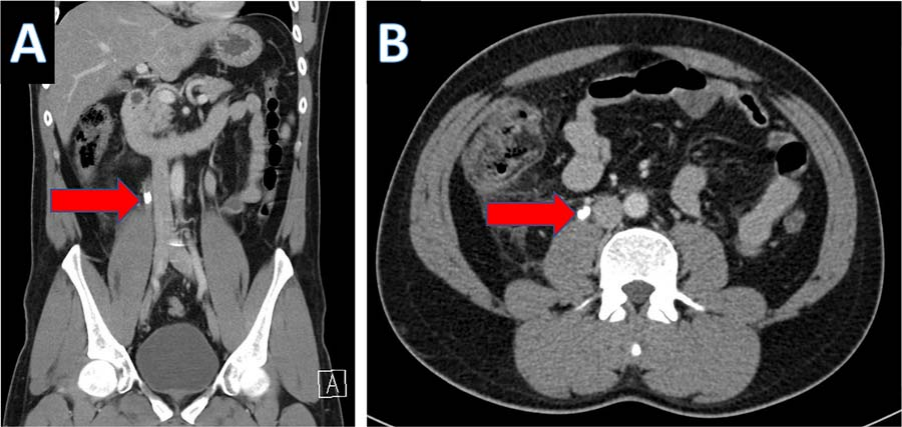
CT scan showing right ureteral calculus (A: coronal; B: axial).

**Figure 2:**
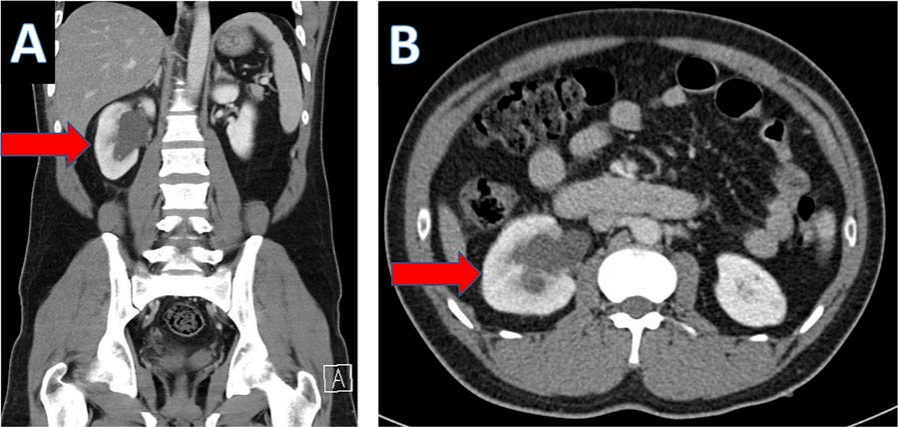
CT scan showing hydronephrosis of the right kidney (A: coronal; B: axial).

An IV contrast-enhanced CT scan of the abdomen and pelvis revealed an 8-mm proximal to mid-ureteral obstructive stone on the right side (Fig. 1), causing severe hydronephrosis of the right kidney and proximal hydroureter (Fig. 2).

**Figure 3:**
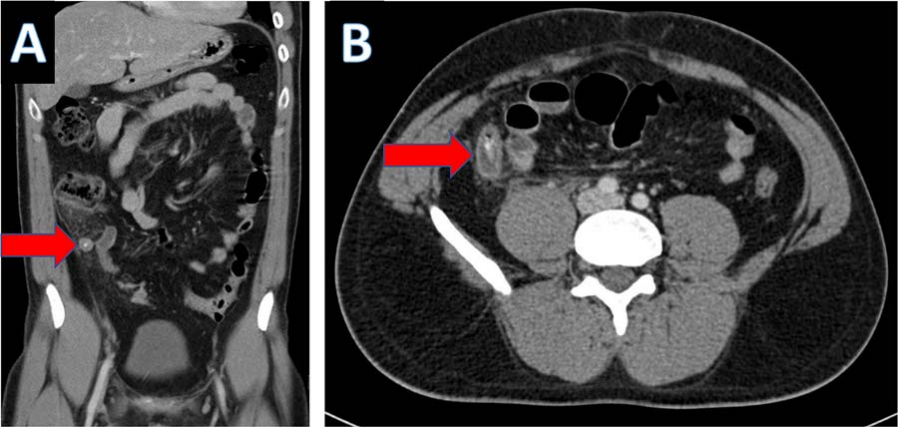
Coronal CT scan showing appendicolith (A) and axial CT showing appendicitis (B).

Multiple tiny obstructive stones were also noted bilaterally. The appendix measured approximately 1.4 cm in maximum caliber, with an appendicolith present within the appendiceal lumen (Fig. 3a). Significant surrounding fat stranding was noted around the appendix, and multiple enlarged lymph nodes with signs of reactive inflammation were seen adjacent to the cecum (Fig. 3b).

The cardiologist was consulted regarding the patient's elevated blood pressure and recommended pain control. The urologist was also consulted and advised on the observation of creatinine levels. The plan was an admission of the patient with NPO, IV fluids, antibiotics, analgesia, PPI, valsartan 80 mg STAT, and to undergo an urgent laparoscopic appendectomy with right ureteroscopy and double-J stent insertion. The patient underwent the surgery on the same day successfully, and the intraoperative findings revealed an acutely inflamed appendix with the presence of exudates, but no evidence of perforation, abscess formation, or gangrene, and the base appeared healthy. A postoperative KUB was performed the next morning, revealing that the right double-J catheter was appropriately placed. The patient was doing well on the first postoperative day with no complaints and was discharged with a course of ciprofloxacin. Out-patient follow-up appointments were scheduled with the urologist, cardiologist, and general surgeon. The resected appendix specimen was sent for histopathological examination, which later revealed focal mucosal ulcerations with transmural neutrophilic infiltration. The inflammatory process extended through the serosa and into the mesoappendiceal fat with no evidence of atypia or malignancy observed.

## Discussion

Our patient presented with appendicitis, with incidental findings of a right ureteral stone causing severe hydroureteronephrosis. The chief complaint in the present case was right lower quadrant abdominal pain, which was suggestive of acute appendicitis. Similar to acute appendicitis, ureterolithiasis also provokes colicky abdominal pain. However, in most cases, when acute appendicitis is diagnosed initially, ureterolithiasis is often overlooked in emergency care. Therefore, the concurrent presence of both these disease conditions has been rarely described in the literature. A literature review revealed only a few similar cases (Table 2). This case highlights the importance of maintaining a high index of suspicion for the coexistence of multiple pathologies in patients with RLQ abdominal pain. While scoring systems like the Alvarado Score can be valuable diagnostic tools, they may not reliably predict the presence of concurrent pathologies. CT scanning has become the gold standard diagnostic modality for evaluating right lower quadrant abdominal pain, with high sensitivity and specificity for acute appendicitis and nephrolithiasis [10–13]. This makes CT the preferred approach when multiple acute pathologies in the right lower abdomen are suspected [14]. The management of patients with concurrent acute appendicitis and obstructive ureterolithiasis typically involves a multidisciplinary approach involving general surgeons and urologists. In most cases, surgical intervention is warranted. Laparoscopic appendectomy is the standard treatment for acute appendicitis, while ureteroscopy with stone extraction and stent placement is the preferred treatment for obstructive ureterolithiasis. In our case, both acute pathologies were managed appropriately through prompt surgical interventions. Our treatment approach is in line with other cases of concurrent disease processes reported in the literature.

**Table 2:** Review of literature.

Study	Year	Country	Chief complaint at presentation	R/L	HN/HU (Yes/No)	Laboratory and temperature	Imaging studies	Age	Sex	Stone size
Vagianos *et al.* [2]	2000	Greece	Abd. pain, high fever	R	Yes	Temp: 102.2°F; WBC:17; UA: microscopic hematuria	US and CT with contrast	75	Male	__
Lang *et al.* [3]	2005	USA	Abd. pain, vomiting, gross hematuria	R	No	Temp: 98.4°F; WBC:12.600; neutrophils: 90%; UA: 20 RBCs; INR: 0.92	Spiral CT	37	Male	4.6 mm
Kwon *et al.* [4]	2007	South Korea	Abd. pain, nausea, vomiting, then anuria after appendectomy	R	Yes	WBC: 14.98; Cr: 265.2μmol/L; UA: microscopic hematuria (20–30 HPF)	US & nonenhanced CT	20	Female	__
Spiel *et al.* [5]	2012	USA	Abd. pain	R	Yes	Temp: 97.2 ° F; WBC: 10.3; Cr: 1.9 mg/dL; BUN: 31 mg/dL	CT with contrast	47	Male	6 mm
Anjum *et al.* [6]	2012	UK	Abd. pain, nausea, diarrhea, dysuria	L	Yes	WBC: 14.3; CRP: 150 mg/L; UA: microscopic hematuria (2+ RBCs)	Nonenhanced CT	12	Male	12 mm
Mestrinho *et al.* [7]	2015	Brazil	Abd. pain, dysuria, adynamia	R	No	UA: microscopic hematuria	CT with contrast	21	Male	10 mm
Daniel *et al.* [8]	2018	USA	Abd. pain, subjective fevers, chills, nausea	R	Yes	WBC: 12.3; neutrophils:76%; UA: significant trace leukocyte esterase	CT with contrast	34	Male	6.5 × 3.5 mm
Hiraoka *et al.* [9]	2022	Japan	Abd. pain, vomiting	R	Yes	Temp: 99.68° F; WBC: 8.37; neutrophils:77.8%, CRP: 0.36 mg/dL; UA: microscopic hematuria (3 + RBCs/WBCs: 1–4 HPF)	CT	12	Female	4.2 mm
Our case										

List of abbreviations: Temp = Temperature; Cr = Creatinine; Abd. = abdominal; R/L = right or left ureteral stone; HN = hydronephrosis; HU=Hydroureter; UA = Urinanalysis; US=Ultrasound. WBCs are expressed in 10^9^/L.

## Conclusions

The diagnosis of abdominal pain can often be achieved through a thorough clinical examination supported by laboratory tests, sometimes eliminating the need for further imaging studies. However, this approach may not be suitable for evaluating patients with right lower abdominal pain, as it may fail to predict the synchronous presentation of multiple pathologies. This case underscores the need for a comprehensive workup, including appropriate imaging, when evaluating patients with RLQ abdominal pain. Though such presentations are uncommon, clinicians should maintain a high index of suspicion for concurrent conditions.

## Data Availability

The data used to support the findings of this study are included in the article.
